# Supplementary material for: Association between oral microbiome and seven types of cancers in East Asian population: a two-sample Mendelian randomization analysis
Source: Front Mol Biosci. 2023 Nov 21;10:1327893. doi: 10.3389/fmolb.2023.1327893 (PMC10702768; doi:10.3389/fmolb.2023.1327893)
Supplement: Supplementary file 1 [file DataSheet1.ZIP › Supplementary Materials/MR plots of saliva/saliva═╝/Breast cancer/pheno.1026_to_breast cancer_forest.pdf]

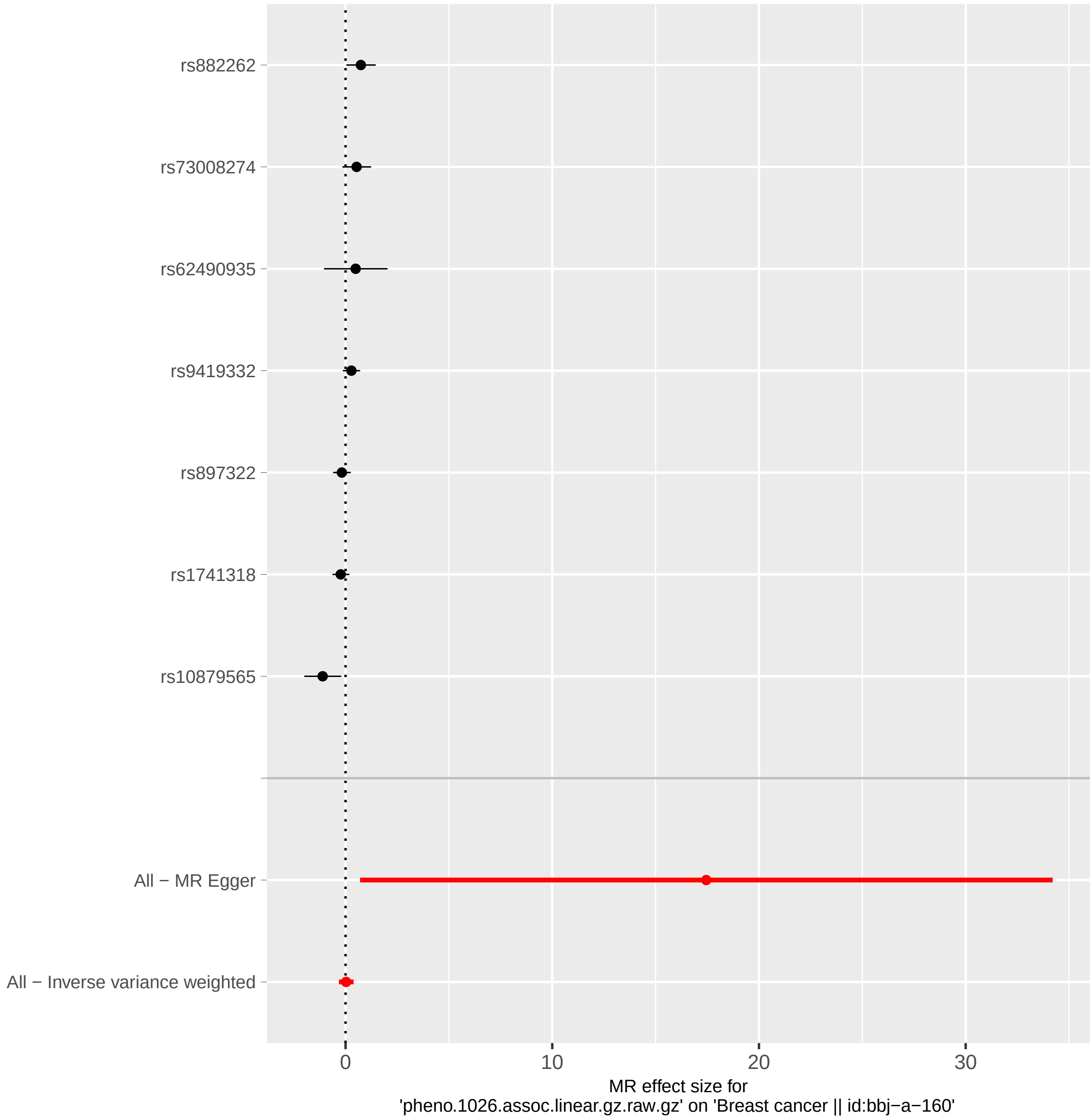
rs882262
rs73008274
rs62490935
rs9419332
rs897322
rs1741318
rs10879565
All − MR Egger
All − Inverse variance weighted
0
10
20
30
MR effect size for
'pheno.1026.assoc.linear.gz.raw.gz' on 'Breast cancer || id:bbj−a−160'
